# Exploring anabasine excretion factor in individuals who use tobacco cigarettes: a preliminary estimate

**DOI:** 10.1093/ntr/ntag042

**Published:** 2026-02-23

**Authors:** Min-Tz Weng, Qiuda Zheng, Yao Deng, Shakti Shrestha, Wenqing Fan, Phong K Thai, Coral E Gartner, Zhe Wang, Kathryn J Steadman

**Affiliations:** School of Pharmacy and Pharmaceutical Sciences, The University of Queensland, Brisbane, Queensland 4102, Australia; Queensland Alliance for Environmental Health Sciences, The University of Queensland, Brisbane, Queensland 4102, Australia; School of Pharmacy and Pharmaceutical Sciences, The University of Queensland, Brisbane, Queensland 4102, Australia; School of Pharmacy and Pharmaceutical Sciences, The University of Queensland, Brisbane, Queensland 4102, Australia; School of Pharmacy and Pharmaceutical Sciences, The University of Queensland, Brisbane, Queensland 4102, Australia; Queensland Alliance for Environmental Health Sciences, The University of Queensland, Brisbane, Queensland 4102, Australia; School of Public Health, The University of Queensland, Brisbane, Queensland 4006, Australia; Queensland Alliance for Environmental Health Sciences, The University of Queensland, Brisbane, Queensland 4102, Australia; School of Pharmacy and Pharmaceutical Sciences, The University of Queensland, Brisbane, Queensland 4102, Australia

**Keywords:** anabasine, conventional cigarettes, nicotine vaping products, excretion, nicotine, smoking

## Abstract

**Introduction:**

Population-level models predicting tobacco use would benefit from inclusion of an accurate anabasine excretion factor. This study aimed to explore the excretion of anabasine in people who use conventional cigarettes (CCs) and nicotine vaping products (NVPs).

**Methods:**

A total of 72 participants were enrolled: 22 people who smoked CCs, 20 people who used NVPs, and 30 people who had never smoked or vaped. The quantity of CC and NVP use was documented over a 3-day period. Composite 24-hour urine samples were collected on day 3 and analyzed using LC–MS/MS to quantify nicotine, cotinine (COT), 3-hydroxycotinine (3HC), anabasine, and anatabine. The anabasine excretion factor was calculated for urine samples containing anabasine.

**Results:**

Nicotine exposure, as the molar sum of nicotine and its metabolites (COT and 3HC) was higher for the NVP group compared to the CC group (*p* = 0.0460). Anabasine concentrations were low in urine of the NVP group but the difference from the CC group did not reach statistical significance (*p* = 0.0646). Data from 19 individuals in the CC group were used to calculate anabasine excretion factor, giving a value of 9.02%.

**Conclusions:**

The excretion factor for anabasine was calculated from 24-hour urine samples for 19 individuals who smoked cigarettes, providing a preliminary estimate that may be incorporated into predictive modeling for population-level tobacco product use. Given the small sample size of this study, future research with larger cohorts is required to provide more reliable estimates.

**Implications:**

Individuals in this study who used NVPs had higher exposure to nicotine than those who smoked CCs. The excretion factor for anabasine was calculated, which may support the estimation of tobacco use at the population level via anabasine load in wastewater samples.

## Introduction

Monitoring tobacco and nicotine consumption at the population level is crucial for understanding the efficacy of tobacco control policies from a public health perspective.^1^ Wastewater-based epidemiology (WBE) is considered to be a valuable method for monitoring substance use^2^ including tobacco use within populations.^3^^,^^4^ Most WBE studies so far involve analyzing the levels of key nicotine metabolites, cotinine (COT) and 3-hydroxycotinine (3HC) in wastewater and converting them into nicotine levels and then back-calculating to estimated tobacco consumption.^5^

However, the increasing prevalence of non-tobacco nicotine-containing products raises a challenge in accurately measuring tobacco via measuring nicotine’s metabolites as these products also contain nicotine.^6^ Depending solely on COT and 3HC levels for estimation can result in an overestimation of tobacco consumption as people who use non-smoked nicotine products (e.g., nicotine vaping products [NVPs] and nicotine replacement therapies [NRTs]) also excrete nicotine metabolites into wastewater.^7–9^ Therefore, it is crucial to identify biomarkers that distinguish between tobacco and non-tobacco nicotine sources for WBE to accurately estimate tobacco consumption from other nicotine use at the population level.

Anabasine and anatabine have been suggested as biomarkers for distinguishing between people who smoke and those who use non-smoked nicotine because these minor tobacco alkaloids are much more prominent in tobacco leaves than in purified nicotine products such as NRTs or NVPs.^9^ For example, a urinary cut-off for anatabine and anabasine (2 ng/mL) has been reported to differentiate between individuals who smoke and those using pharmaceutical-grade nicotine contained in NRT.^10–12^ However, for WBE, anatabine is considered not suitable because it is found in some non-tobacco food and supplements,^13^ and its content in cigarettes is more variable than anabasine.^7^ Meanwhile anabasine has been tested and used in several WBE studies to indicate the relative proportion of tobacco use compared to total nicotine consumption at the population level.^7–9^^,^^14^^,^^15^ While anabasine shows promise as a biomarker for estimating tobacco consumption, limited pharmacokinetic data hinder the calculation of its excretion factor. A recent study relied on extrapolation from a single urine sample provided by each of 10 individuals to calculate the average excretion of anabasine from each cigarette.^7^ While this is a useful guide, a more comprehensive analysis is necessary to accurately calculate the excretion factor for anabasine from both conventional cigarettes (CCs) and NVPs use.

The present study aimed to calculate the excretion factor for anabasine using 24-hour urine samples and compare anabasine levels between individuals who exclusively smoke, vape, or do not use nicotine-containing products.

## Methods

This study was approved by the University of Queensland Human Research Ethics Committee (2021/HE002652).

### Study design

This study was a prospective observation study. Individuals who exclusively smoked CCs at least once a day, individuals who exclusively used NVPs at least once a day, and individuals who had never smoked or vaped were recruited. Participants were required to be aged 18 years old or above, have sufficient English ability to understand the participant information sheet and instructions, absence of regular cannabis use, commitment to refrain from using other tobacco products throughout the data collection period, willing to record a 3-day diary and collect all urine produced for 24 hours on day 3, and be able to attend the study center in Brisbane to collect and return the study materials.

### Study procedure and data collection

The study was conducted between March and August 2022. It was advertised via posters, flyers, word of mouth, and social media. Participants completed a survey about their demographic profile (sex, age, level of education, work status, and ethnicity), and details of the tobacco and vape products that they regularly use. Details of nicotine-containing products, including the brand, strength, and the flavor of the product, the number of puffs or cigarettes that they consumed over the 3 days was collected in a 3-day diary (File S1). On the third day, all urine produced over a 24-hour period was collected in a 3 L container. Immediately following the final urine collection (the first urine passed in the morning of day 4), the urine container was returned for processing. People who had never smoked or vaped completed the demographic survey and the 24-hour urine collection at their convenient time. Participants were advised to store the collection bottle in a cool place, refrigerated if possible, during the sample collection period.^16^

### Urine sample handling and analysis

The volume of urine for individual participants was measured. Samples were aliquoted and stored in a −20°C freezer until analysis. Before analysis, urine samples were diluted with ultrapure water (100 times for nicotine and its metabolites, and 5 times for anabasine and anatabine), followed by addition of internal standards, treatment with β-glucuronidase (*Helix pomatia*, Type HP-2, aqueous solution, ≥ 100 000 units/mL, Sigma-Aldrich, Darmstadt, Germany), and passing through a 0.2 μm filter (4 mm diameter, 0.2 μm pore size, Agilent, Mulgrave, Australia). The pretreated urine samples were analyzed using liquid chromatography coupled with tandem mass spectrometry (LC–MS/MS) for quantification of nicotine, its metabolites (COT and 3HC), and minor tobacco alkaloids, anabasine and anatabine.^14^ The limits of detection (LOD) for anabasine, anatabine, nicotine, COT, and 3HC were 0.1, 0.05, 0.1, 0.01, and 0.02 μg/L, respectively. The corresponding limits of quantification (LOQ) were 0.2, 0.15, 0.2, 0.02, and 0.05 μg/L, respectively.

The creatinine (urinary) colorimetric assay (Cayman Biosciences, Michigan, USA) was used in accordance with manufacturer instructions for the measurement of creatinine levels.^17^

### Data analysis

#### Estimation of nicotine intake from self-reported use data

Nicotine intake from cigarettes (mg/day) was calculated using the average number of cigarettes smoked per day (CPD) across the 3 days recorded in the daily diary and assuming the nicotine content inhaled per cigarette to be 0.9 mg^8^ in the following equation:


$${\begin{align*}& \mathrm{Nicotine}\ \mathrm{intake}\ \left(\mathrm{mg}/\mathrm{day}\right)=\mathrm{0.9}\ \mathrm{}\mathrm{mg}/\mathrm{cig}\times 3\ \mathrm{day}\ \mathrm{average}\ \mathrm{CPD}\ \left(\mathrm{cig}/\mathrm{day}\right) \end{align*}}$$

Nicotine intake from NVPs (mg/day) was calculated using participant self-reported product details (nicotine strength (%), volume and number of puffs in the container) and use. The number of puffs per day (PPD) was the average across the 3 days recorded in the daily diary. If the information provided by the participant did not include nicotine content, and it was not possible to identify the nicotine content of their product on the internet, the average nicotine content of all available commercial devices in this study was used to predict the nicotine content per puff. If participants provided liquid volume use, it was converted to PPD using device information available on the internet. The nicotine intake for NVPs was calculated as follows:


$${\begin{align*} \mathrm{Nicotine}\ \mathrm{intake}\ \left(\mathrm{mg}/\mathrm{day}\right)&=\frac{\mathrm{Product}\ \mathrm{nicotine}\ \mathrm{content}\ \left(\mathrm{mg}\right)\ }{\mathrm{Product}\ \mathrm{content}\mathrm{s}\ \left(\mathrm{puff}\mathrm{s}\right)}\times3\ \mathrm{day}\ \mathrm{average}\ \mathrm{PPD}\ \left(\mathrm{puff}/\mathrm{day}\right) \end{align*}}$$

#### Calculation of concentrations in urine

The concentration of nicotine, COT, 3HC, and minor tobacco alkaloid levels were measured after glucuronide deconjugation using glucuronidase, and as such the reported values represent the total of the free alkaloid and its glucuronide conjugate. Using these values, nicotine exposure (μmol/L) was expressed as urine total nicotine equivalents, which was calculated as the molar sum of nicotine and 5 metabolites (NE6), that is, nicotine + COT + 3HC + nicotine-glucuronide + COT-glucuronide + 3HC-glucuronide. If the concentration of any alkaloid was below LOD, the value was replaced with LOD/2. If the concentration was between LOD and LOQ, the value was replaced by LOQ/2.^18^ The concentration of each alkaloid was also normalized to creatinine concentration using individual concentrations divided by their respective creatinine concentrations to account for variation in hydration status of our participants.

#### Anabasine excretion factor

Calculation of the excretion factor for anabasine (EF_anabasine_) was based on the study of Zheng et al. (2023).^8^ To account for variation in tobacco weight in cigarettes, EF_anabasine_ was calculated as a percentage instead of μg/cigarette. The excretion factor for anabasine (%) was calculated according to the following equation:


$${\begin{align*} {\mathrm{EF}}_{\mathrm{anabasine}}\left(\%\right)&=\frac{{\mathrm{C}}_{\mathrm{anabasine}}\!\times \!{\mathrm{MW}}_{\mathrm{anabasine}}\!\times \!{\mathrm{EF}}_{\mathrm{C}\mathrm{OT}\!+\!3\mathrm{HC}}\!\times\! {\mathrm{Ab}}_{\mathrm{nicotine}}}{\left({C}_{\mathrm{C}\mathrm{OT}}+{C}_{3\mathrm{HC}}\right)\times{\mathrm{MW}}_{\mathrm{nicotine}}\times{\mathrm{Ab}}_{\mathrm{anabasine}}}\times 100\% \end{align*}}$$

where *C* represents concentration (nmol/mg cr), and MW denotes molecular weight (g/mol). Ab_nicotine_ refers to the amount of nicotine absorbed from one cigarette, estimated as 0.9 mg,^8^ and Ab_anabasine_ indicates the amount of anabasine absorbed from one cigarette, estimated as 0.0072 mg due to an inhalation ratio of 0.008 between anabasine and nicotine.^7^ The calculation was performed using the established excretion factor of both COT and 3HC as the comparator with the value of EF_COT + 3HC_ = 72.12% from the literature.^8^

### Statistical analysis

Cigarette use (CPD) in the CC group and NVP use (PPD) in the NVP group were compared across 3 days using a one-way ANOVA. Following confirmation of normality, urine output was compared among the CC, NVP, and non-use groups using a one-way ANOVA. Following log transformation of chemical concentrations (minor tobacco alkaloids, nicotine, COT, 3HC, and NE6 in urine samples) to improve normality, a one-way ANOVA was conducted to compare the CC, NVP, and non-use groups, with Tukey post-hoc comparisons. A simple linear regression between estimated nicotine intake and measured nicotine exposure (NE6) was investigated among the CC and NVP groups. All statistical analyses were conducted using GraphPad Prism version 10.0 (GraphPad Software, Boston, MA).

## Results

### Demographics

**Figure 1:**
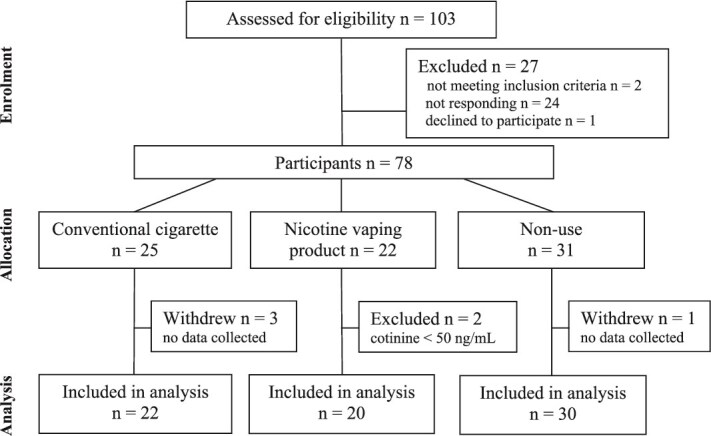
The progression of participants through this study.

A total of 78 individuals provided informed consent to participate. Following the withdrawal of 4 participants, 74 individuals collected data (Figure 1). Two participants who identified as using NVPs were excluded from analysis due to their urinary cotinine levels being below 50 ng/mL, which was a criterion for both the CC and NVP groups.^12^ Among the 72 participants included in the analysis, 22 exclusively smoked CCs, 20 exclusively used NVPs, and 30 had never smoked or vaped. The majority of participants were male (71%), White (57%), aged between 18 and 29 (63%), and were currently studying or had university level education (Table 1). The number of CPD used in the CC group and PPD in the NVP group showed no difference across the 3 days (CPD *p* = 0.9403 and PPD *p* = 0.5725). No difference in 24-hour urine volume was observed across 3 groups (*p* = 0.1270; Table 2).

**Table 1:** Participant demographics and their daily use of nicotine products as an average across 3 days grouped according to use of conventional cigarettes (CCs), nicotine vaping products (NVPs), or no use of nicotine products (non-use) (*n* = 72).

Category	CC (*n* = 22)		NVP (*n* = 20)		Non-use (*n* = 30)		Total (*n* = 72)	
	*n*	%	*n*	%	*n*	%	*n*	%
Gender								
Male	19	86	12	60	20	67	51	71
Female	3	14	8	40	10	33	21	29
Age (year)								
18–29	10	45	18	90	17	57	45	63
30–39	9	41	0	0	10	33	19	26
40 or above	3	14	2	10	3	10	8	11
Ethnicity								
Asian	7	32	8	40	12	40	27	38
Indigenous	1	5	0	0	0	0	1	1
Latino	1	5	2	10	0	0	3	4
White	13	59	10	50	18	60	41	57
Conventional cigarette—average number smoked per day (CPD) from day 1 to day 3								
1–5	11	50	-	-	-	-	-	-
6–10	4	18	-	-	-	-	-	-
11–15	5	23	-	-	-	-	-	-
16–20	0	0	-	-	-	-	-	-
20 or above	2	9	-	-	-	-	-	-
Nicotine vaping product—average number of puffs per day (PPD) from day 1 to day 3								
1–20	-	-	1	5	-	-	-	-
21–40	-	-	3	15	-	-	-	-
41–60	-	-	7	35	-	-	-	-
61–80	-	-	5	25	-	-	-	-
80 or above	-	-	4	20	-	-	-	-

- represents not applicable.

**Table 2:** Volume of urine collected in the 24-hour period (mL) and concentration (nmol/mg creatinine) of nicotine, cotinine (COT), 3-hydroxycotinine (3HC), anabasine, and anatabine measured in the urine (*n* = 72) according to use of conventional cigarettes (CCs), nicotine vaping products (NVPs), or no use of nicotine products (non-use). For each individual chemical, the mean, SD, median, and range of values are provided; means sharing the same letter are not significantly different, while a different letter after the mean indicates significant difference (*p* < 0.05).

	CC (*n* = 22)				NVP (*n* = 20)				Non-use (*n* = 30)			
Chemical (nmol/mg cr)	Mean	SD	Median	Range	Mean	SD	Median	Range	Mean	SD	Median	Range
Anabasine	0.013^a^	0.017	0.0068	0.0006–0.078	0.0082^a^	0.014	0.0026	0.0001–0.047	0.0018^b^	0.0051	0.0005	0.0001–0.028
Anatabine	0.019^a^	0.028	0.01	0.0003–0.13	0.0058^b^	0.013	0.00085	0.0001–0.054	0.00033^c^	0.00047	0.0002	0.0001–0.0027
Nicotine	3.96^a^	4.18	2.31	0.42–17.36	5.29^a^	3.54	4.35	0.62–11.46	0.0014^b^	0.0010	0.0012	0.0002–0.0041
COT	6.60^a^	5.99	5.10	0.44–22.09	11.58^a^	8.25	8.44	1.55–34.33	0.0019^b^	0.0078	0.0004	0.00001–0.043
3HC	7.73^a^	8.15	4.85	0.16–35.83	17.55^a^	15.52	14.08	1.06–58.53	0.0078^b^	0.037	0.0001	0.00002–0.206
Urine output (mL)	1659	757.7	1545	400–3055	1758	676.7	1830	700–3000	2037^$^	623.5^$^	2020	800–3000

$ Missing urine volume data for 1 participant.

### Alkaloid quantities

All chemical concentrations showed significant differences among the CC, the NVP, and the non-use groups (*p* < 0.01). Levels of all alkaloids in the urine of individuals who identified as not using nicotine were exceptionally low, and consequently were significantly lower than that of both CC and NVP groups (Table 2).

The minor tobacco alkaloid, anatabine, was significantly higher in the urine of the CC group than the NVP group (*p* < 0.001). Anabasine concentration trended toward being higher in the CC group than in the NVP group, although the difference did not reach statistical significance (*p* = 0.0646).

**Figure 2:**
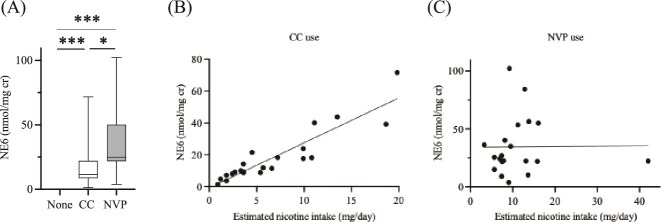
(A) Box and whisker plots of nicotine exposure (NE6, nmol/mg cr) in 24-hour urine samples among non-use, CC, and NVP groups showing the median, the 25th and 75th percentiles and the range. The lines above the plot link pairs for which the mean values are significantly different. The scatter plots show estimated daily nicotine intake (mg/day) plotted against measured nicotine exposure (NE6) for (B) the CC group (*r*^2^ *=* 0.8253; *p* < 0.001) and (C) the NVP group (*r*^2^ = 0.0001; *p* = 0.9655). NE6 is the sum of the molar concentration of nicotine, COT, 3HC, nicotine-glucuronide, COT-glucuronide, 3HC-glucuronide expressed in nmol/mg cr. Abbreviations: CC, conventional cigarette; COT, cotinine, 3HC, 3-hydroxycotinine; NPVs, nicotine vaping products.

Concentrations of nicotine and its metabolites did not differ between the CC and the NVP groups when assessed individually (nicotine *p* = 0.2401; COT *p* = 0.0889; 3HC *p* = 0.0979). However, when assessed together as NE6, the NVP group was higher (34.42 ± 25.02 nmol/mg cr) than the CC group (18.29 ± 16.75 nmol/mg cr) (*p* = 0.0460). There was considerable variation in NE6 between individuals, ranging from 3.9 to 102.2 nmol/mg cr for the NVP group, and from 1.4 to 71.7 nmol/mg cr for the CC group (Figure 2A).

There was a strong relationship between NE6 and nicotine intake estimated from self-reported cigarette use data (*r*^2^ = 0.8253; *p* < 0.001), indicating that self-reported cigarette use (CPD) provides a good estimate of nicotine exposure (Figure 2B). However, in the case of NVP use, the lack of a correlation between estimated nicotine intake and NE6 (*r*^2^ = 0.0001; *p* = 0.9655) shows that our estimate of nicotine intake as PPD using self-reported vape use was a poor measure of nicotine exposure (Figure 2C).

### Anabasine excretion factor

**Figure 3:**
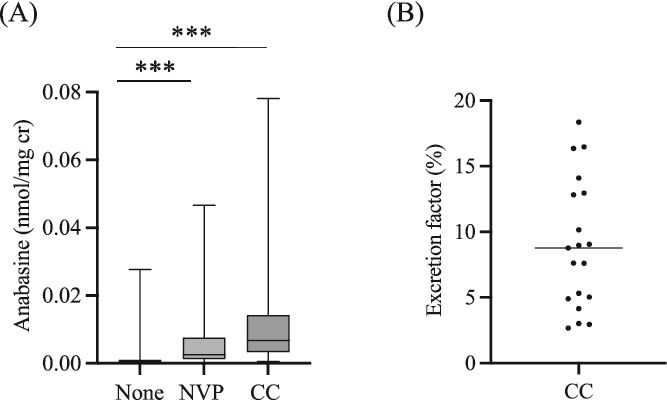
(A) Box and whisker plots of anabasine levels (nmol/mg cr) showing the median, the 25th and 75th percentiles, and the range. Data are shown for 24-hour urine samples among non-use (*n* = 30), NVP (*n* = 20), and CC (*n* = 22) groups. (B) The excretion factor for anabasine (%) in the CC group for individuals with greater than 0.002 nmol/mg cr urinary anabasine (*n* = 19), ranging from 2.67% to 18.36% with an average of 9.02%. Abbreviations: CC, conventional cigarette; NPVs, nicotine vaping products.

Anabasine concentrations among the non-use, the NVP, and the CC groups are presented in Figure 3A. Anabasine concentrations were above 0.002 nmol/mg cr in 19 individuals within the CC group, and were regarded as suitable for use in the calculation of an anabasine excretion factor as the concentrations were above the LOQ and the average cigarette consumption was greater than one cigarette. When calculated using the COT + 3HC excretion factor of 72.12%^8^ as the basis, the average excretion factor for anabasine was 9.02% ± 4.95% (Figure 3B).

Concentrations exceeded 0.002 nmol/mg cr in 3 individuals from the non-use group and 12 from the NVP group. One participant in the NVP group reported using a tobacco-flavored e-liquid; this person had the highest anabasine concentration within the group (0.047 nmol/mg cr). However, we were unable to estimate the expected quantity of anabasine inhaled from NVPs due to the inhalation ratio between anabasine and nicotine from NVPs being unknown. Therefore, it was not possible to calculate an excretion factor for this group.

## Discussion

The excretion factor for anabasine was calculated to be 9.02% in our study. We used 24-hour urine samples from 19 individuals who use CCs to calculate the anabasine excretion factor, which represents the excretion of anabasine as a percentage of the estimated amount of anabasine absorbed. Previously the level of anabasine excreted into urine samples was presented as the quantity excreted per cigarette.^7^ One advantage of using the percentage is that it remains consistent even if the amount of anabasine in tobacco products varies, and secondly this approach aligns with the excretion factors in the literature for nicotine metabolites, that is, COT + 3HC are 72.12%.^8^ However, given the small sample size and limited participant demographics of this study, this finding should be considered as a preliminary estimate. Future studies with larger sample sizes are needed to provide more reliable estimates.

Several factors could affect the accuracy of the excretion factor calculation. Firstly, the estimation of anabasine absorption in our equation was derived from a single study using a smoking machine.^19^ Further validation of this parameter across different populations and tobacco sources would be beneficial. Secondly, our calculation relied on known excretion factors for nicotine metabolites, COT and 3HC. Pharmacokinetic data shows that the excretion factor for COT and 3HC varies significantly, ranging from 23.2% to 38.1% and from 21% to 58.9%, respectively.^20^^,^^21^ We used the value 72.12% for COT + 3HC obtained from the average of 8 studies.^8^ Variations in these excretion factors may stem from differences in excretion among individuals of different ethnic backgrounds. Notably, the excretion factors for COT and 3HC were derived from data primarily involving Caucasian populations,^20^ while our study population was only 60% Caucasian. One-third of our participants were Asian, who are more likely to metabolize nicotine via the non-CYP2A6 pathway, resulting in lower levels of COT and 3HC in urine^20^^,^^22^ and thus lower excretion factors compared to Caucasian participants.^20^ Therefore, it is important to recognize that the excretion factor for anabasine calculated in this study, 9.02%, is an average value and interindividual variation remains unaccounted for and not fully understood.

In our study, analysis of NE6 indicated that participants who vaped nicotine products exhibited higher nicotine exposure than the participants who smoked cigarettes. While the relationship between nicotine exposure (NE6) and self-reported cigarette use was strong, there was no relationship between nicotine exposure and our calculation of nicotine intake based on self-reported NVP use. The nicotine content of each puff was calculated from device details and use patterns recorded by participants. However, differences in puff duration and volume combined with device characteristics influence nicotine output and absorption from NVP products.^23–27^ For example, people who are experienced in NVP use tend to inhale for longer durations compared to beginners or people who smoke cigarettes which can lead to greater nicotine absorption.^23^^,^^27^ Hence, rather than relying solely on self-reported use, it would have been preferable to accurately determine the total volume of e-liquid consumed.^28^

In addition, several studies have identified the mislabeling of nicotine content on NVPs.^29–31^ For example, the majority of products fell within a 30% range of the label claim, whereas in some products the specified nicotine content exceeded 50%.^31^ Given the inconsistent content of nicotine, conducting a direct product analysis of nicotine concentration becomes imperative to minimize discrepancies in estimating nicotine intake from NVPs. Therefore, with much higher level of uncertainties from puff length and nicotine content of NVPs, self-reported data cannot be used as a predictor of nicotine intake among our NVP group.

### Limitations

Within the sample of participants who smoked and vaped, participants tended toward the lower end of cigarette and vape use, as their urinary concentrations of alkaloids were predominantly clustered at the lower end of the range, with fewer individuals exhibiting mid-range to high-range values. While 2 ng/mL anabasine in urine has been reported to differentiate between cigarette and NRT use, only 5 participants in the CC group reached this level. In fact, 3 participants in the NVP group and 1 participant in the non-use group had urine anabasine levels greater than 2 ng/mL, making it unclear whether this originated from their vape fluid or unreported cigarette use. Some participants who reported extensive NVP use had minimal urinary nicotine (Figure 2), which might be attributed to differences in inhalation technique leading to variations in nicotine intake.^23^^,^^24^^,^^26^^,^^27^ Without validating the number of cigarettes smoked and NVP device and e-liquid use through testing the product strength (nicotine content) and anabasine levels, it is possible that participant documentation did not accurately reflect the products being used. This study did not consider previous cigarette use among people who vaped, so there may have been potential for anabasine contamination. However, none of those in the NVP group reported also using tobacco products in their baseline survey, and only 2 of those in the CC group reported co-use of vapes. The potential for contamination was further minimized by the 3-day study design. The half-lives of nicotine, its metabolites (COT and 3HC), and minor tobacco alkaloids (anabasine and anatabine) are approximately 2, 16, 6, 16, and 10 hours, respectively.^10^^,^^12^ Given the long half-lives of cotinine and anabasine, the washout period is estimated to be approximately 2.7–3.3 days (equivalent to 4–5 half-lives). Saliva cotinine and carbon monoxide testing prior to the study may have helped to confirm current cigarette or NVP use.^32^

Greater than 30% of individuals had urinary concentrations below LOQ for anatabine in the NVP group, and for nicotine, 3HC, anatabine, and anabasine in the non-use group (File S2). When a substantial proportion of values are estimated rather than directly measured, summary statistics do not accurately reflect true variability. Consequently, the robustness of group comparisons is diminished, and the results should be interpreted with caution.

Since anabasine has been measured in some NVPs,^29^ its contribution to wastewater load from vaping should be considered. However, the proportion of anabasine to nicotine in NVPs is approximately 12–3000 times lower than that found in cigarettes.^7^^,^^33^^,^^34^ With the growing prevalence of vaping and the variability in anabasine content across NVPs, future studies should take this potential source into consideration.

### Conclusion

In conclusion, this study provides the excretion factor of anabasine (9%), which has been calculated from 24-hour urine samples from 19 individuals who smoke tobacco cigarettes. Applying the excretion factor for anabasine may aid in estimating cigarette consumption in wastewater, providing insights into population smoking rates. Further studies with larger and diverse populations are required to obtain more reliable estimates.

## Supplementary Material

Supplementary_file_1_12Nov2025_ntag042

Supplementary_file_2_12Nov2025_ntag042

## Data Availability

Data will be provided on request to the authors.
