# Supplementary material for: Exploring anabasine excretion factor in individuals who use tobacco cigarettes: a preliminary estimate
Source: Nicotine Tob Res. 2026 Feb 23;28(8):1357–64. doi: 10.1093/ntr/ntag042 (PMC13389531; doi:10.1093/ntr/ntag042)
Supplement: Supplementary_file_1_12Nov2025_ntag042 [file supplementary_file_1_12nov2025_ntag042.pdf]

Baseline questionnaire

Participant ID Code:_______________

Note: These questions can be completed online following completion of the consent form in a Checkbox survey format using drop-down options, tick boxes, and free text fields.

There are six sections, please complete all of the questions.

1. Demographic details

1a. How old are you? ……… years

1b. What is your assigned or biological sex?

☐ Female ☐ Male ☐ Indeterminate/other

1c. What is the highest level of education you have completed?

☐ No formal schooling
☐ Less than primary school
☐ Primary school completed
☐ Junior secondary school completed
☐ Senior high school completed
☐ College/University completed
☐ Post graduate degree

1d. Thinking about the place you usually live, are there any other people living in your home who smoke tobacco daily?

☐ Yes ☐ No (Go to 1f)

1e. Not including yourself, how many people living in your home currently smoke tobacco daily? _____________

1f. Which of the following best describes your main work status over the past 12 months?

☐ Employed
☐ Self-employed
☐ Non-paid volunteer
☐ Student
☐ Homemaker
☐ Retired
☐ Not in paid work

1g. What is your ethnic background? (Select all that apply)

- ☐ Oceanian (*Australian Peoples* - Australian, Australian Aboriginal, Australian South Sea Islander, Torres Strait Islander; *New Zealand Peoples* – Maori, New Zealander; *Melanesian and Papuan* – New Caledonian, Ni-Vanuata, Papua New Guinean, Solomon Islander, Melanesian and Papuan nec; *Microneasian* – I-Kiribati, Nauruan, Micronesian nec; *Polynesian* – Cook Islander, Fijian, Niuean, Samoan, Tongan, Hawaiian, Tahitian, Tokelauan, Tuvaluan, Pitcairn, Polynesian nec)
- ☐ North-West European (*British* – English, Scottish, Welsh, Channel Islander, Manx, British nec; *Irish* – Irish; *Western European* - Austrian, Dutch, Flemish, French, German, Swiss, Belgian, Frisian, Luxembourg, Western European nec; *Northern European* – Danish, Finnish, Icelandic, Norwegian, Swedish, Northern European nec)
- ☐ Southern and Eastern European (*Southern European* - Basque, Catalan, Italian, Maltese, Portuguese, Spanish, Gibraltarian, Southern European nec; *South Eastern European* – Albanian, Bosnian, Croatian, Greek, Macedonia, Moldovan, Montenegrin, Romanian, Roma Gypsy, Serbian, Solvene, Cypriot, Vlach, South Eastern European nec; *Eastern European* – Belarusan, Czech, Estonian, Hungarian, Latvian, Lithuanian, Polish, Russian, Slovak, Ukrainian, Sorb/Wend, Eastern European nec)
- ☐ North African and Middle Eastern (*Arab* – Algerian, Eygptian Iraqi, Jordanian, Kuwaiti, Lebanese, Libyan, Moroccan, Palestinian, Saudi Arabian, Syrian, Tunisian, Yemeni, Bahraini, Emirati, Omani, Qatari, Arab nec; *Jewish* - Jewish, *Peoples of the Sudan* – Bari, Darfur, Dinka, Nuer, South Sudanese, Sudanese, People of the Sudan nec; *Other North African and Middle Eastern* – Berber, Coptic, Iranian, Kurdish, Turkish, Assyrian, Chaldean, Mandaean, Nubian, Yezidi, Other North African and Middle Eastern nec)
- ☐ South-East Asia (*Mainland South-East Asian* – Anglo-Burmese, Burmese, Hmong, Khmer (Cambodian), Lao, Thai, Veitnamese, Karen, Mon, Chin, Rohingya, Mainland South-East Asian nec; *Maritime South-East Asian* – Filipino, Indonesian, Javanese, Madurese, Malay, Sudanese, Timorese, Acehnese, Balinese, Bruneian, Kadazan, Singaporean, Temoq, Maritime South-East Asian nec)

- ☐ North-East Asia (Chinese Asian – Chinese, Taiwanese, Chinese Asian nec; *Other North-East Asian* – Japanese, Korean, Mongolian, Tibetan, Other North-East Asian nec)
- ☐ Southern and Central Asia (*Southern Asian* – Anglo-Indian, Benagli, Burgher, Gujarati, Indian, Malayali, Nepalese, Pakistani, Pubjabi, Sikh, Sinhalese, Maldivian, Bangladeshi, Bhutanese, Fijian Indian, Kashmiri, Parsi, Sindhi, Sri Lankan, Sri Lankan Tamil, Indian Tamil, Tamil nfd, Telugu, Southern Asian nec; *Central Asian* – Afghan, Armenian, Georgian, Kazakh, Pathan, Uzbek, Azeri, Hazara, Tajik, Tatar, Turkmen, Uighur, Kyrgyz, Central Asian nec)
- ☐ People of the Americas (*North American* – African American, American, Canadian, French Canadian, Hispanic North American, Native North American Indian, Bermudan, North American nec; *South American* – Argentinian, Bolivian, Brazilian, Chilean, Colombian, Ecuadorian, Guyanese, Peruvian, Uruguayan, Venezuelan, Paraguayan, South American nec; *Central American* – Mexican, Nicaraguan, Salvadoran, Costa Rican, Guatemalan, Mayan, Central American nec; *Caribbean Islander* – Cuban, Jamaican, Trinidadian Tobagonian, Barbadian, Puerto Rican, Caribbean Islander nec)
- ☐ Sub-Saharan African (*Central and Western African* – Akan, Fulani, Ghanaian, Nigerian, Yoruba, Ivorean, Liberian, Sierra Leonean, Acholi, Cameroonian, Congolese, Gio, Igbo, Krahn, Mandinka, Senegalese, Themne, Togolese, Central and Western African nec; *Southern and East African* – Afrikaner, Angolan, Eritrean, Ethiopian, Kenyan, Malawian, Mauritian, Mozambican, Namibian, Oromo, Seychellois, Somali, South African, Tanzanian, Ugandan, Zambian, Zimbabwean, Amhara, Batswana, Hutu, Masai, Tigrayah, Tigre, Zulu, Burundian, Kunama, Madi, Ogaden, Rwandan, Shona, Swahili, Swazilander, Southern and East African nec)

2. Questions on nicotine product(s) and use

Please complete the table below by filling in the information in each column for each product that you currently use regularly. Each row is a different type of product.

	**Name of the product (tick if you regularly use this product)**		**Brands / strength**	**Frequency**	**Used since** (approximate if not sure)
1	☐ Manufactured cigarettes			____ Cigarettes per day/week/month	
2	☐ Roll-your-own cigarettes			____ Cigarettes per day/week/month	
3	☐ Other smoked product	☐ Cigars		____ Cigars per day/week/month	
4		☐ Water pipe with tobacco?		____ per day/week/month	
		☐			
5	☐ Electronic cigarette (select all that applies to you)	☐ I usually use nicotine in the e-cigarette		___ Puffs per day/week/month<br>• Which strength of liquid nicotine do you use mostly? ____mg/mL<br>• How long does a 10mL vial of nicotine containing e-cig liquid last for you? _______days	
		☐ I don't usually use nicotine in the e-cigarette		___ Puffs per day/week/month	

		Do you use e-liquids that contain natural tobacco extract? (if yes what is the brand?)<br>☐ Yes<br>☐ No<br>☐ Don’t know			
6	☐ Nicotine replacement therapy (NRT) products	☐ Chewing gums		_____ per day/week/month	
7		☐ Lozenges		_____ per day/week/month	
8		☐ Patches		_____ per day/week/month	
9		☐ Mouth sprays		_____ Puffs per day/week/month	
10		☐ Inhalators		_____ Puffs per day/week/month	
11	☐ Heated tobacco products (e.g. IQOS)			_____ per day/week/month	
12	☐ Oral tobacco (e.g. snus, pituri)			_____ per day/week/month	

THE UNIVERSITY OF QUEENSLAND AUSTRALIA

Three-day diary

Instructions:

- For three days in a row write down all of the nicotine products that you use (e.g., if you start on Monday as day 1, record all products used from when you wake up on Monday morning until you wake up on Thursday morning).
- On day 3, collect all your urine (pee) in the large brown container provided.
- On day 3 phone XXXX XXX XXX to arrange for the return of this diary and your 24-hour urine container as soon after you finish the final collection as possible on day 4.
- Please use only one type of nicotine or tobacco product for the 3 days. For example, if you usually smoke and vape, please select one item only to use for these three days.
- Do not consume grapefruit juice during the 3-day period as it interferes with the metabolism of nicotine.

Note: to avoid needing to carry the large container with you, we recommend that you schedule the three days so that day 3 falls on a day that you can be at home all day and night.

Collecting your urine

It is important that the urine collection is completed over a 24-hour period. It is recommended that you start the collection process in the morning when you first get up.

If you forget to use the bottle during the collection period, contact the research team on XXXX XXX XXX. Contact the research team for advice if you cannot complete the collection for any reason.

1. At the beginning of day three, empty your bladder into the toilet and mark the time on day 3 of this diary.
2. Over the next 24 hours collect all of your urine in the bottle.
   - Use the beaker to collect your urine every time you pee and pour the urine into the large container.
   - Keep the large container closed except from when you pour urine into it.
3. The last collection (on day 4) should be as near as possible to the time of the first discarded pee on day 3 and should include the urine you pass first thing on day 4
4. Make sure that the start and finish date and time are clearly marked on the bottle.
5. Keep the collection bottle in a cool place during the collection period, refrigerate if possible. We recommend keeping the beaker beside the toilet and placing a note on the toilet to remind yourself to use the beaker rather than the toilet for urine.

Participant ID:___________

Day 1

Date:

Record your nicotine use below.

Product brand/strength:

Flavour (if applicable):

Time of day used nicotine or tobacco product	Quantity/amount used (e.g. 1 cigarette, number of puffs of e-cigarette, 1x lozenge etc)

If you have anything to add please write below:

The University of Queensland
AUSTRALIA

Day 2

Participant ID:___________

Date:

Record your nicotine use below.

Product brand/strength:

Flavour (if applicable):

**Time of day used nicotine or tobacco product**	**Quantity/amount used (e.g. 1 cigarette, number of puffs of e-cigarette, 1x lozenge etc)**

If you have anything to add please write below:

The University of Queensland
AUSTRALIA

Day 3

Participant ID:___________

Collect all of your urine today

Date: **Urine collection start time:**

Record your nicotine use below.

Product brand/strength:

Flavour (if applicable):

**Time of day used nicotine or tobacco product**	**Quantity/amount used (e.g. 1 cigarette, number of puffs of e-cigarette, 1x lozenge etc)**

If you have anything to add please write below:

Remember to phone to arrange return of this diary and your 24-hour urine container tomorrow.
