# Supplementary material for: Exploring anabasine excretion factor in individuals who use tobacco cigarettes: a preliminary estimate
Source: Nicotine Tob Res. 2026 Feb 23;28(8):1357–64. doi: 10.1093/ntr/ntag042 (PMC13389531; doi:10.1093/ntr/ntag042)
Supplement: Supplementary_file_2_12Nov2025_ntag042 [file supplementary_file_2_12nov2025_ntag042.pdf]

Weng M-T, Zheng Q, Deng Y, Shrestha S, Fan W, Thai PK, Gartner CE, Wang Z, Steadman KJ (2025) Exploring anabasine excretion factor in individuals who use tobacco cigarettes: a preliminary estimate. Nicotine and Tobacco Research.

Supplementary file 2A. Concentration of components measured in 24-hour urine samples from participants in the study who used combustible cigarettes (CC). Values that were below LOD were assigned the value LOD/2 (blue font), and values that were between LOD and LOQ were assigned the value LOQ/2 (green font). All other values were greater than LOQ (black font).

		Concentration after de-conjuncation (nmol/mg cr)						
Group	Sample code	Anabasine	Anatabine	Nicotine	Cotinine	Hydroxycotinine	Anabasine + anatabine	Nicotine + cotinine + hydroxycotinine
CC	C_01	0.0127	0.0183	0.988	3.087	5.017	0.0310	9.092
	C_04	0.0041	0.0043	4.405	5.531	1.827	0.0084	11.763
	C_05	0.0068	0.0052	2.941	7.531	7.149	0.0120	17.621
	C_06	0.0068	0.0099	2.257	3.492	3.539	0.0167	9.288
	C_07	0.0168	0.0211	5.813	5.306	2.955	0.0379	14.074
	C_08	0.0063	0.0098	1.338	1.962	1.529	0.0161	4.829
	C_09	0.0006	0.0005	2.868	5.328	3.156	0.0011	11.351
	C_10	0.0274	0.0456	6.875	16.277	16.114	0.0730	39.266
	C_11	0.0035	0.0041	2.421	3.514	2.849	0.0075	8.784
	C_13	0.0134	0.0142	2.343	6.013	9.875	0.0276	18.231
	C_14	0.0218	0.0294	2.282	6.836	14.853	0.0511	23.971
	C_15	0.0076	0.0119	1.855	2.840	2.484	0.0195	7.180
	C_17	0.0095	0.0172	12.000	4.889	4.678	0.0268	21.567
	C_18	0.0009	0.0003	0.762	0.442	0.157	0.0011	1.361
	C_19	0.0043	0.0053	1.270	1.558	0.784	0.0096	3.612
	C_20	0.0781	0.1253	17.358	18.500	35.832	0.2034	71.689
	C_21	0.0116	0.0161	5.482	15.583	19.020	0.0277	40.084
	C_31	0.0018	0.0032	1.751	7.693	8.654	0.0050	18.098
	C_32	0.0025	0.0028	0.421	2.040	5.592	0.0053	8.053
	C_33	0.0028	0.0032	0.469	2.250	7.342	0.0061	10.061
	C_34	0.0038	0.0043	2.211	2.509	3.988	0.0082	8.707
	C_36	0.0393	0.0595	8.921	22.085	12.739	0.0987	43.745

Supplementary file 2B. Concentration of components measured in 24-hour urine samples from participants in the study who used nicotine vaping products (NVP). Values that were below LOD were assigned the value LOD/2 (blue font), and values that were between LOD and LOQ were assigned the value LOQ/2 (green font). All other values were greater than LOQ (black font).

		Concentration after de-conjuncation (nmol/mg cr)						
Group	Sample code	Anabasine	Anatabine	Nicotine	Cotinine	Hydroxycotinine	Anabasine + anatabine	Nicotine + cotinine + hydroxycotinine
NVP	V_01	0.0071	0.0060	2.199	2.982	5.166	0.0131	10.346
	V_02	0.0004	0.0002	1.242	1.552	1.061	0.0006	3.855
	V_03	0.0002	0.0001	2.982	6.853	12.433	0.0003	22.267
	V_04	0.0001	0.0001	0.619	3.328	5.215	0.0002	9.162
	V_05	0.0466	0.0543	9.923	18.189	11.934	0.1009	40.046
	V_06	0.0058	0.0002	5.715	13.641	34.264	0.0061	53.619
	V_07	0.0014	0.0004	6.548	14.752	14.850	0.0018	36.150
	V_08	0.0078	0.0003	10.519	19.175	54.458	0.0081	84.152
	V_09	0.0032	0.0021	2.826	8.374	15.889	0.0053	27.089
	V_10	0.0026	0.0022	2.865	8.352	14.228	0.0048	25.444
	V_11	0.0089	0.0064	9.746	8.514	3.959	0.0153	22.218
	V_12	0.0018	0.0001	5.750	10.718	5.285	0.0019	21.754
	V_13	0.0018	0.0001	7.600	9.746	17.604	0.0018	34.951
	V_15	0.0430	0.0214	7.575	20.312	28.441	0.0643	56.328
	V_16	0.0028	0.0022	2.396	7.600	14.263	0.0050	24.259
	V_17	0.0026	0.0021	2.316	7.141	13.080	0.0047	22.537
	V_21	0.0237	0.0159	9.335	34.326	58.534	0.0396	102.195
	V_22	0.0026	0.0010	11.461	24.850	18.743	0.0035	55.054
	V_23	0.0003	0.0002	2.374	4.972	7.648	0.0005	14.994
	V_24	0.0012	0.0007	1.850	6.197	13.939	0.0019	21.986

Supplementary file 2C. Concentration of components measured in 24-hour urine samples from participants in the study who identifed as not using any nicotine products at all. Values that were below LOD were assigned the value LOD/2 (blue font), and values that were between LOD and LOQ were assigned the value LOQ/2 (green font). All other values were greater than LOQ (black font).

Group	Sample code	Concentration after de-conjuncation (nmol/mg cr)						
		Anabasine	Anatabine	Nicotine	Cotinine	Hydroxycotinine	Anabasine + anatabine	Nicotine + cotinine + hydroxycotinine
Non-use	N_01	0.00083	0.00042	0.00083	0.00015	0.00014	0.00125	0.00112
	N_02	0.00042	0.00021	0.00176	0.00032	0.00007	0.00064	0.00216
	N_03	0.00846	0.00019	0.00272	0.00097	0.00006	0.00864	0.00375
	N_04	0.00032	0.00016	0.00064	0.00017	0.00005	0.00048	0.00087
	N_05	0.02768	0.00020	0.00040	0.00088	0.00007	0.02788	0.00134
	N_07	0.00036	0.00018	0.00036	0.00037	0.00006	0.00054	0.00079
	N_08	0.00013	0.00007	0.00094	0.00007	0.00002	0.00020	0.00103
	N_09	0.00042	0.00021	0.00216	0.00156	0.00862	0.00063	0.01234
	N_10	0.00059	0.00030	0.00311	0.00068	0.00010	0.00089	0.00390
	N_11	0.00066	0.00034	0.00066	0.00046	0.00011	0.00100	0.00124
	N_12	0.00121	0.00061	0.00121	0.00064	0.00020	0.00182	0.00205
	N_13	0.00069	0.00035	0.00069	0.00071	0.00012	0.00104	0.00152
	N_14	0.00137	0.00014	0.00027	0.00014	0.00370	0.00151	0.00411
	N_15	0.00112	0.00028	0.00056	0.00010	0.00009	0.00140	0.00076
	N_16	0.00058	0.00029	0.00058	0.00009	0.00010	0.00087	0.00076
	N_17	0.00036	0.00018	0.00155	0.00047	0.00286	0.00055	0.00488
	N_18	0.00047	0.00024	0.00081	0.00018	0.00242	0.00071	0.00342
	N_19	0.00049	0.00074	0.00049	0.00091	0.00124	0.00122	0.00263
	N_20	0.00012	0.00006	0.00313	0.00146	0.00242	0.00018	0.00700
	N_21	0.00021	0.00011	0.00210	0.00115	0.00004	0.00032	0.00329
	N_22	0.00022	0.00011	0.00022	0.00022	0.00004	0.00034	0.00048
	N_23	0.00076	0.00039	0.00324	0.00036	0.00013	0.00115	0.00373
	N_24	0.00096	0.00010	0.00121	0.00002	0.00194	0.00106	0.00317
	N_25	0.00219	0.00016	0.00171	0.00066	0.00300	0.00235	0.00537
	N_26	0.00018	0.00009	0.00199	0.00032	0.00003	0.00027	0.00234
	N_27	0.00013	0.00007	0.00142	0.00001	0.00002	0.00020	0.00146
	N_28	0.00203	0.00051	0.00102	0.00009	0.00163	0.00255	0.00274
	N_29	0.00041	0.00021	0.00409	0.04287	0.20614	0.00062	0.25309
	N_30	0.00035	0.00265	0.00142	0.00016	0.00006	0.00300	0.00164
	N_31	0.00054	0.00027	0.00109	0.00112	0.00009	0.00082	0.00230

Weng M-T, Zheng Q, Deng Y, Shrestha S, Fan W, Thai PK, Gartner CE, Wang Z, Steadman KJ (2025) Exploring anabasine excretion factor in individuals who use tobacco cigarettes: a preliminary estimate. Nicotine and Tobacco Research.

Supplementary file 2D. Excretion factor calculated for each of the individuals who used combustible cigarettes (CC group). Values were not calculated for three individuals for whom urinary anabasine concentrations were below 0.002 nmol/mg cr.

Group	Sample code	Anabasine excretion factor (%)
CC	C_01	14.1
	C_04	5.0
	C_05	4.2
	C_06	8.8
	C_07	18.4
	C_08	16.4
	C_09	-
	C_10	7.6
	C_11	4.9
	C_13	7.6
	C_14	9.0
	C_15	12.8
	C_17	9.0
	C_18	-
	C_19	16.5
	C_20	13.0
	C_21	3.0
	C_31	-
	C_32	3.0
	C_33	2.7
	C_34	5.3
	C_36	10.2
